# Supplementary material for: Single-Cell Proteomic Analysis Dissects the Complexity of Tumor Microenvironment in Muscle Invasive Bladder Cancer
Source: Cancers (Basel). 2021 Oct 29;13(21):5440. doi: 10.3390/cancers13215440 (PMC8582554; doi:10.3390/cancers13215440)
Supplement: Supplementary file 1 [file cancers-13-05440-s001.zip › cancers-1402777-supplementary.pdf]

cancers

Article

Single-Cell Proteomic Analysis Dissects the Complexity of Tumor Microenvironment in Muscle Invasive Bladder Cancer

Chao Feng [1,2,3,†], Xi Wang [1,2,4,†], Yuting Tao [1,2,3,†], Yuanliang Xie [1,2,5], Zhiyong Lai [1,2], Zhijian Li [1,2], Jiaxin Hu [1,2], Shaomei Tang [1,2], Lixin Pan [1,2], Liangyu He [1,2,6,7], Qiuyan Wang [1,2,4,*], Tianyu Li [1,2,6,7,*] and Zengnan Mo [1,2,6,7]

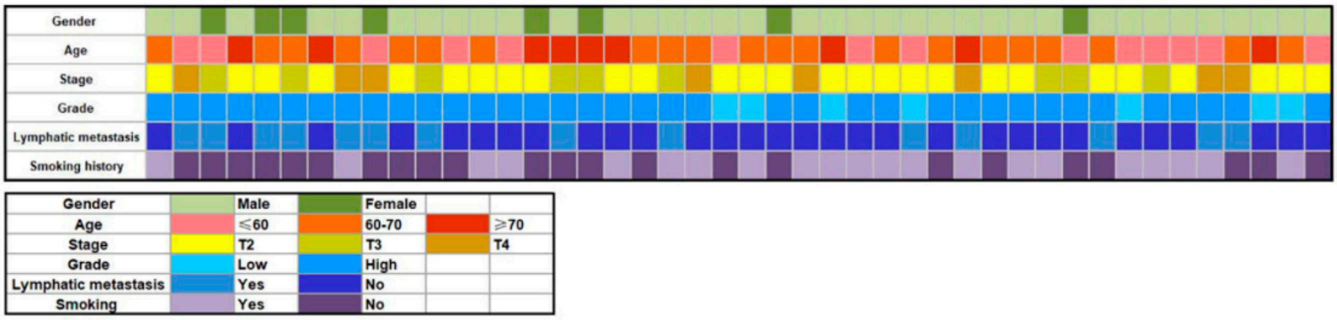

Figure S1. Detail information of samples. Clinical information of samples.

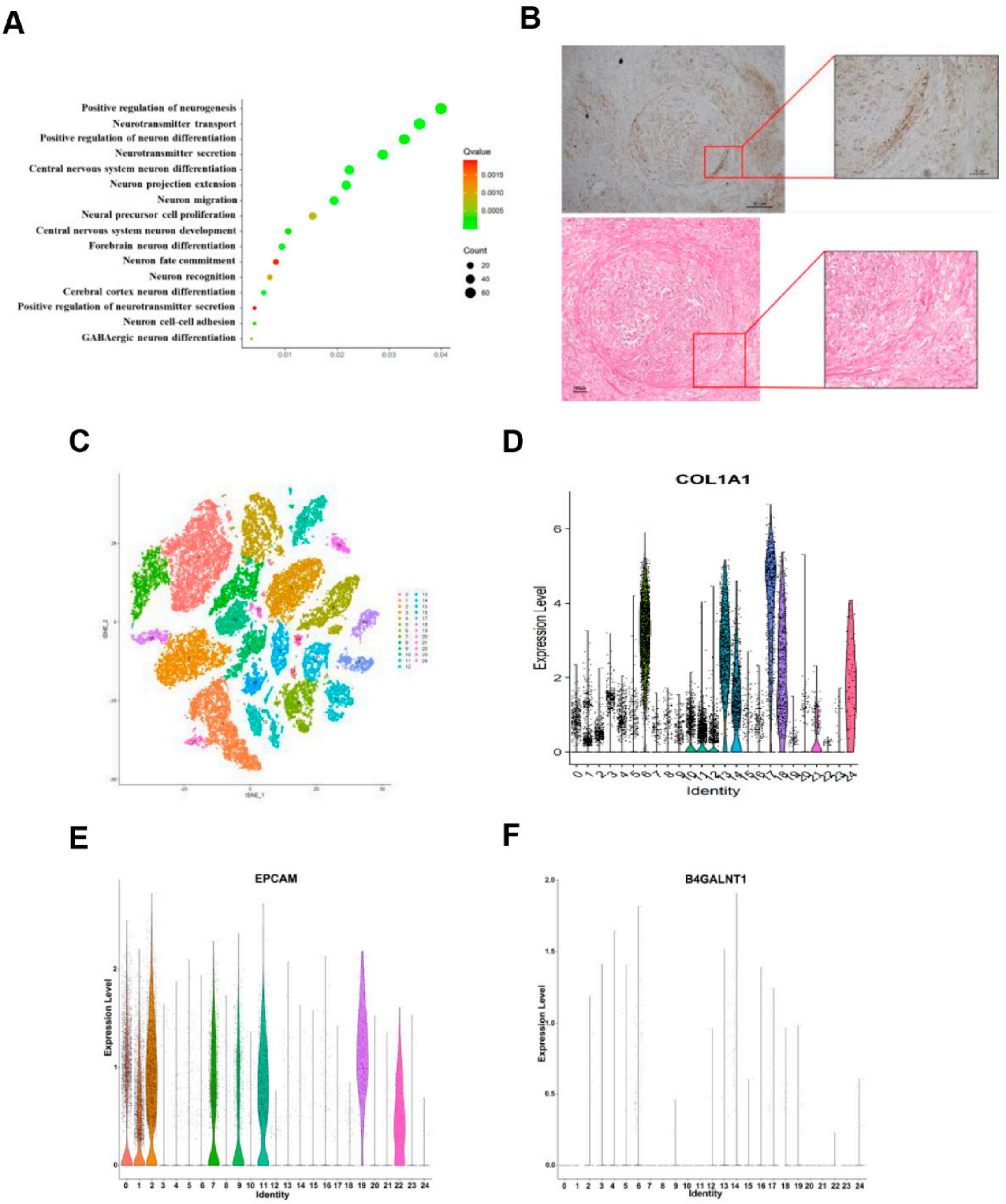

Figure S2. Detail characterization of cluster 6 high- and low-abundance group patients. (A) GO analysis shows that the genes significantly relate to B4GALNT1 expression are enriched in neuro-relative pathways. (B) IHC staining images of B4GALNT1 and H&E staining images of the same area. Scale bar as showed in images. (C) t-SNE clustering of all single cells from 8 bladder cancer tissues. (D-F) Expression levels of COL1A1 (D), EPCAM (E) and B4GALNT1 (F) in the 25 clusters.

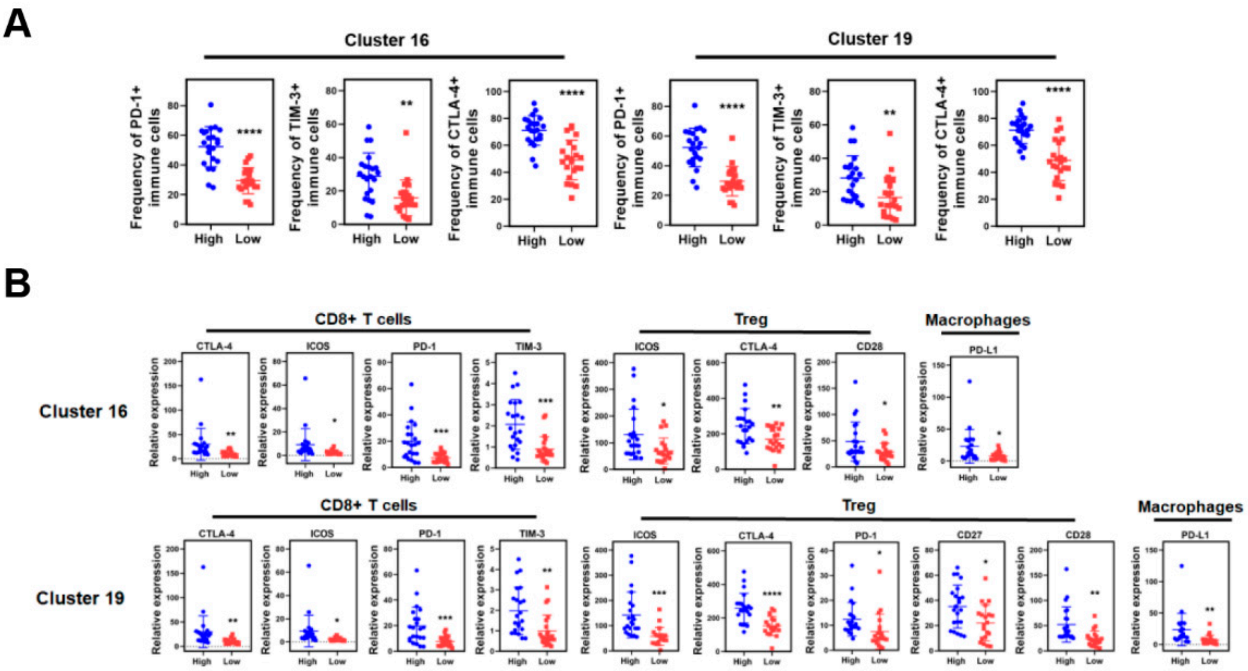

Figure S3. Detail characterization of MIBC immune microenvironment. (A) Frequencies of PD-1⁺, TIM-3⁺, CTLA-4⁺ immune cells in cluster 16 or 19 high- and low-abundance groups. (B) Expression levels of specific markers of CD8⁺ T cell cluster, Treg and macrophage in cluster 16 or 19 high- and low-abundance groups.

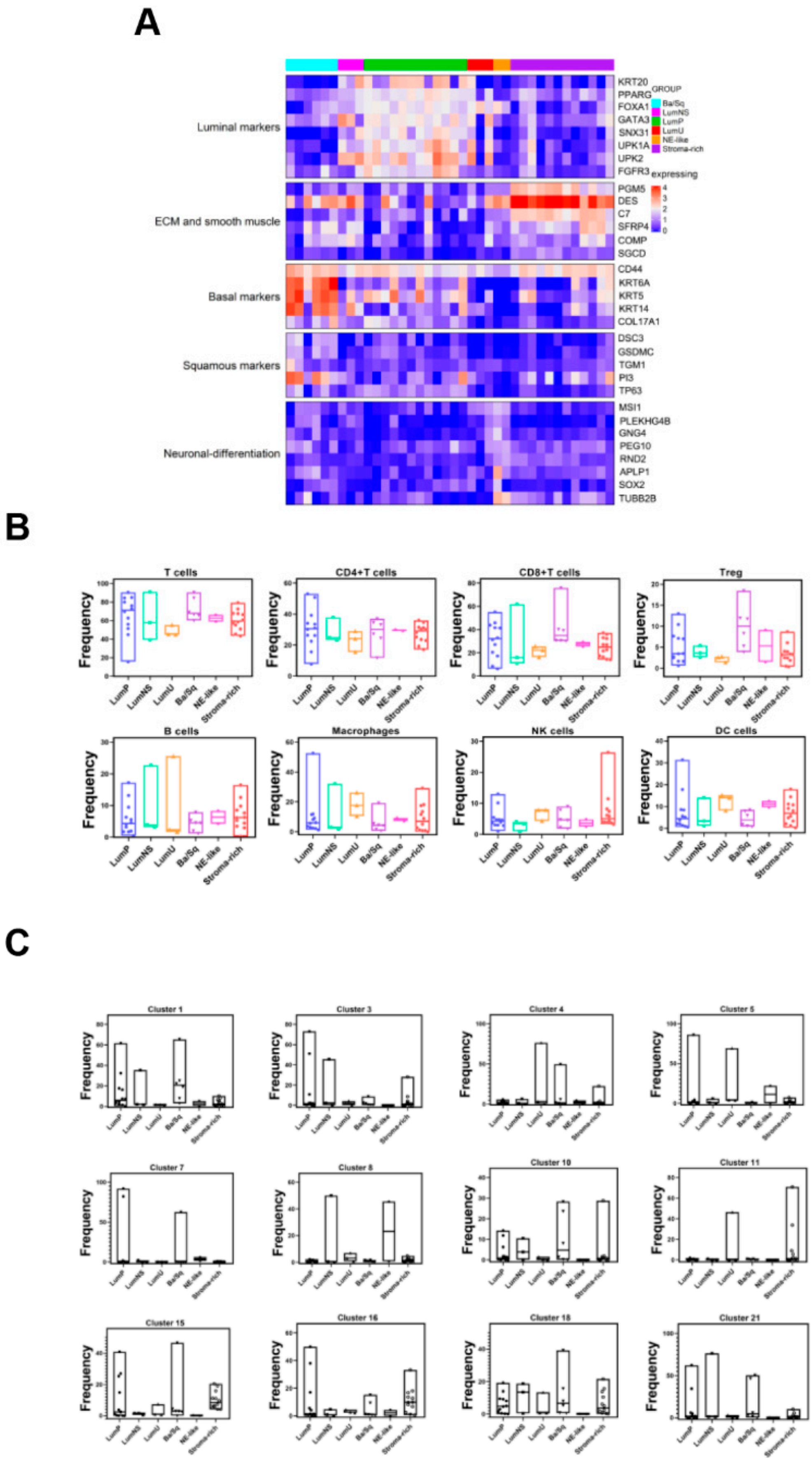
A
Luminal markers
ECM and smooth muscle
Basal markers
Squamous markers
Neuronal-differentiation
KRT20
PPARG
FOXA1
GATA3
SNX31
UPK1A
UPK2
FGFR3
PGM5
DES
C7
SFRP4
COMP
SGCD
CD44
KRT6A
KRT5
KRT14
COL17A1
DSC3
GSDMC
TGM1
PI3
TP63
MSI1
PLEKHG4B
GNG4
PEG10
RND2
APLP1
SOX2
TUBB2B
GROUP
Ba/Sq
LumNS
LumP
LumU
NE-like
Stroma-rich
expressing
B
T cells
CD4+T cells
CD8+T cells
Treg
B cells
Macrophages
NK cells
DC cells
Frequency
LumP
LumNS
LumU
Ba/Sq
NE-like
Stroma-rich
C
Cluster 1
Cluster 3
Cluster 4
Cluster 5
Cluster 7
Cluster 8
Cluster 10
Cluster 11
Cluster 15
Cluster 16
Cluster 18
Cluster 21
Frequency

Figure S4. Characterization of MIBC molecular subtypes. (A) Heatmap of specific markers expression in the six molecular subtypes. (B and C) Frequencies of immune cells types (B) and tumor cell clusters (C) in the six molecular subtypes.

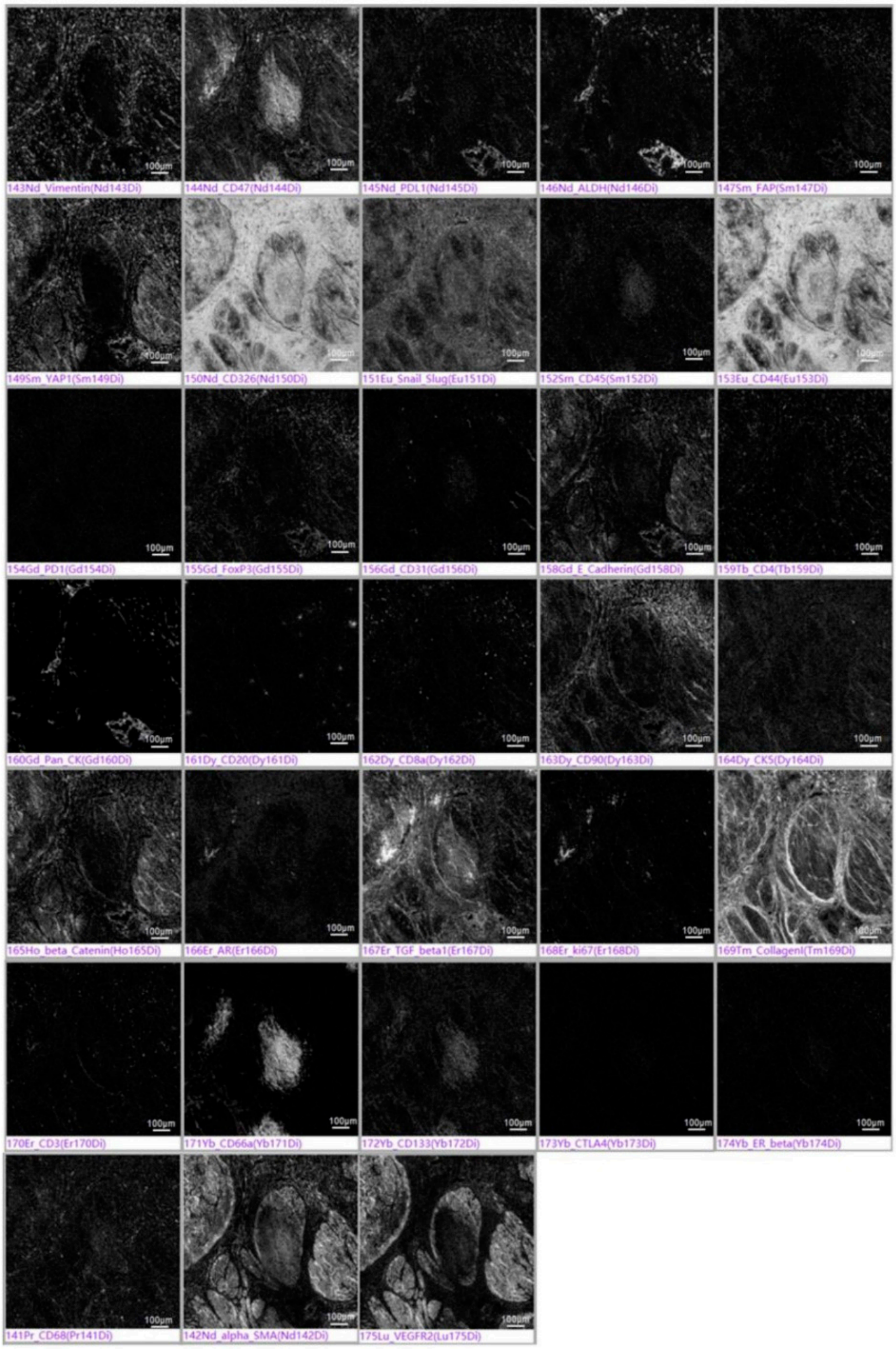

Figure S5. Positive signals of all IMC antibodies from one ROI. Representative IMC images to validate the signals of indicated markers. Scale bar are showed in all images.

Table S1. Purified antibodies of TME panel.

Antibodies	Label /Metal	Clone	Source/ Company	Product_ID /Identifier
CD13	160Gd	WM15	Fluidigm	3160014B
CD24	166Er	ML5	Fluidigm	3166007B
CD34	149Sm	581	Fluidigm	3149013B
CD44	171Yb	IM7	Fluidigm	3171003B
CD47	209Bi	CC2C6	Fluidigm	3209004B
CD54	170Er	HA58	Fluidigm	3170014B
CD90	158Gd	5E10	BioLegend	328102
CD104	173Yb	58XB4	Fluidigm	3173008B
CD133	153Eu	170411	R&D	MAB11331
CD166	145Nd	3A6	BioLegend	343902
CD274 (PD-L1)	175Lu	29E.2A3	Fluidigm	3175017B
CD326 (EpCAM)	141Pr	9C4	Fluidigm	3141006B
CD333 (FGFR3)	151Eu	136334	R&D	MAB766
ALDH	147Sm	44	Fluidigm	3147015B
Androgen Receptor	154Sm	G122-434	Fluidigm	3154018B
c-Myc	176Yb	9E10	Fluidigm	3176012B
CK6	146Nd	EPR4515	Abcam	ab222395
CK5	164Dy	CK5	Abcam	ab53121
Estrogen Receptor -α	142Nd	C-542	Abcam	ab66102
Estrogen Receptor-β	174Yb	14C8	Abcam	ab288
Ki67	161Dy	Ki67	BioLegend	350523
KLF4	162Dy	D1F2	Fluidigm	3162022A
LGR5	155Gd	SA222C5	BioLegend	373802
MET	167Er	D1C2	Fluidigm	3167017A
MUC1	168Er	SM3	Abcam	ab22711
Nanog	169Tm	N31-355	Fluidigm	3169014A
Notch2	165Ho	MHN2-25	Fluidigm	3165026B
OV6	152Sm	OV-6	R&D	MAB2020
p21	159Tb	12D1	Fluidigm	3159026A
p53	143Nd	7F5	Fluidigm	3143018A
Sox2	150Nd	O30-678	Fluidigm	3150019B
Vimentin	156Gd	RV202	Fluidigm	3156023A
CD45	Y89	HI30	BioLegend	304045

Copyright: © 2021 by the authors. Licensee MDPI, Basel, Switzerland. This article is an open access article distributed under the terms and conditions of the Creative Commons Attribution (CC BY) license (http://creativecommons.org/licenses/by/4.0/).

Table S2. Purified antibodies of immune panel.

Antibodies	Label /Metal	Clone	Source/ company	Product_ID /Identifier
CD3	170Er	UCHT1	BioLegend	300402
CD4	176Yb	RPA-T4	BioLegend	300541
CD7	162Dy	CD7-6B7	BioLegend	343111
CD8a	168Er	RPA-T8	BioLegend	301053
CD11b	172Yb	ICRF44	BioLegend	301302
CD11c	160Gd	Bu15	BioLegend	337221
CD14	144Nd	M5E2	BioLegend	301843
CD19	171Yb	HIB19	BioLegend	302247
CD20	142Nd	2H7	BioLegend	302302
CD27	167Er	O323	BioLegend	302839
CD28	152Sm	CD28.2	BioLegend	302937
CD33	163Dy	WM53	BioLegend	303419
CD45	169Tm	HI30	BioLegend	304045
CD45RA	153Eu	HI100	BioLegend	304143
CD45RO	141Pr	UCHL1	BioLegend	304202
CD56	155Gd	5.1H11	BioLegend	362502
CEACAM-6	145Nd	439424	R&D	MAB3934
CD68	175Lu	Y1/82A	BioLegend	333802
CD95	148Nd	DX2	BioLegend	305631
CD127	143Nd	A019D5	BioLegend	351337
CD137	156Gd	4B4-1	BioLegend	309811
CD152 (CTLA-4)	166Er	L3D10	BioLegend	349902
CD163	165Ho	GHI/61	BioLegend	333602
CD274 (PD-L1)	149Sm	29E.2A3	BioLegend	329719
CD278 (ICOS)	151Eu	C398.4A	BioLegend	313502
CD279 (PD-1)	147Sm	EH12.2H7	BioLegend	329941
TIM-3	154Sm	F38-2E2	BioLegend	345019
EOMES	150Nd	644730	R&D	MAB6166
Foxp3	159Tb	PCH101	Thermo	14-4776-82
Granzyme B	164Dy	QA18A28	BioLegend	396402
HLA-DR	174Yb	L243	BioLegend	307651
T-bet	161Dy	4B10	BioLegend	644825
TCR	146Nd	B1	BioLegend	331202
TNF-α	158Gd	MAb11	BioLegend	502902

Table S3. Purified antibodies of IMC pane.

Antibodies	Label /Metal	Clone	Source/ Company	Product_ID /Identifier
FAP	147Sm	DPPIV/CD26	Abcam	ab53066
CD31	156Gd	JC/70A	Abcam	ab9498
VEGFR-2	175Lu	phosphorylation site of tyrosine 951	Abcam	ab39638
E-cadherin	158Gd	24E10	Fluidigm	3158029D
Vimentin	143Nd	RV202	Fluidigm	3143029D
YAP1	149Sm	53-162	Abcam	ab56701
CD66a	171Yb	CD66a-B1.1	Fluidigm	3171020D
Ki-67	168Er	B56	Fluidigm	3168022D
CK-5	164Dy	Cytokeratin 5	Abcam	ab53121
SNAIL+SLUG	151Eu	aa 236-264	Abcam	ab180714
ER-β	174Yb	14C8	Abcam	ab288
CD133	172Yb	aa 848-865	Abcam	ab16518
Foxp3	155Gd	43-100	NOVUS	NB100-39002
PD_L1	145Nd	73-10	Abcam	ab226766
AR	166Er	AR 44	Abcam	ab9474
CTLA4	173Yb	CAL49	Abcam	ab237712
PD-1	154Gd	CAL20	Abcam	ab201811
ALDH	146Nd	44/ALDH	BD	611195
CD47	144Nd	Gln19-Pro139	R&D	AF4670
TGF-β1	167Er	TB21	Abcam	ab190503
CD68	141Pr	KPI	Abcam	ab213096
CD4	159Tb	RPA-T4	Biolegend	300502
CD8a	162Dy	C8/144B	Fluidigm	3162034D
CD20	161Dy	H1	Fluidigm	3161029D
CD3	170Er	C-Terminal	Fluidigm	3170019D
CD45	152Sm	CD45-2B11	Fluidigm	3152016D
Alpha-Smooth Muscle Actin	142Nd	N terminal	Abcam	ab5694
Beta-catenin	165Ho	D13A1	Fluidigm	3165032D
CD90	163Dy	7E1B11	Abcam	ab181469
Collagen I	169Tm	Polyclonal	Fluidigm	3169023D
Pan-CK	160Gd	c-11	Abcam	ab7753
CD326/EpCAM	150Nd	aa 250 to the C-terminus	Abcam	ab71916
CD44	153Eu	692-742	Abcam	ab157107
